# The Effect of Tuberculosis on the Mortality of Cirrhotic Patients: A Population-Based 3-Year Follow-Up Study

**DOI:** 10.1097/MD.0000000000000295

**Published:** 2014-12-02

**Authors:** Tsung-Hsing Hung, Chorng-Jang Lay, Chih-Wei Tseng, Chih-Chun Tsai, Chen-Chi Tsai

**Affiliations:** From the Division of Gastroenterology, Department of Medicine, Dalin Tzu Chi Hospital, Buddhist Tzu Chi Medical Foundation, Chiayi, Taiwan (THH, CWT); School of Medicine, Tzu Chi University, Hualien, Taiwan (THH, CJL, CWT, Chen-Chi Tsai); Division of Infectious diseases, Department of Medicine, Dalin Tzu Chi Hospital, Buddhist Tzu Chi Medical Foundation, Chiayi, Taiwan (CJL, Chen-Chi Tsai); Department of Mathematics, Tamkang University, Tamsui, Taiwan (Chih-Chun Tsai).

## Abstract

Tuberculosis (TB) is a chronic infectious disease caused by *Mycobacterium tuberculosis*. It is still unknown if TB, like other infectious diseases contributes a poor prognosis in cirrhotic patients. The aim of this study was to investigate the impact of TB on the mortality of cirrhotic patients.

National Health Insurance Database, derived from the Taiwan National Health Insurance Program, was used to identify 434 cirrhotic patients with new diagnosis of TB between January 1, 2007 and December 31, 2007. The comparison group consisted of 4340 selected cirrhotic patients without TB in the same period by propensity score matching analysis.

The 30-day, 90-day, 1-year and 3-year mortalities were 10.1%, 24.2%, 43.1%, and 63% in the TB group, and 7.9%, 15.5%, 31.2%, and 53.4% in the non-TB group. After Cox proportional hazard regression model adjusted by the patients’ gender, age, and comorbid disorders, the hazard ratios (HR) in cirrhotic patients with TB for 30-day, 30 to 90-day, 90-day to 1-year, and 1 to 3-year mortalities were 1.33 [95% confidence interval (CI) 0.97–1.83], 1.91 (95% CI 1.45–2.51), 1.46 (95% CI 1.16–1.84), and 1.10 (95% CI 0.88–1.37), compared to the non-TB group.

In conclusion, TB is a risk factor for the mortality of cirrhotic patients. The effect focused on the 30-day to 1-year after diagnosis of TB.

## INTRODUCTION

Cirrhotic patients, regardless of etiology, are prone to have infectious diseases due to an acquired immune deficiency, including antigen-specific and non-specific functions.^1,2^ There are 40% hospitalized cirrhotic patients with infection episodes.^3^ When cirrhotic patients have bacterial infections, they have a 4-fold increase in mortality.^4–6^

Tuberculosis (TB) is an airborne-transmitted infectious disease caused by *Mycobacterium tuberculosis*. TB has been a leading health problem and remains a major cause of death worldwide. It is estimated that one-third of the world's total population are infected with TB bacilli and there are1.2–1.5 million deaths in 2010.^7^ Despite a high degree of medical accessibility, abundant medical resources and the implementation of Direct Observation Therapy/Short course program, Taiwan remains a TB endemic area.

More than 10% of TB cases died during the follow-up period. However, most cases of death during TB treatment in developed countries are often because of causes other than TB.^8,9^ In previous reports, the TB patients with chronic liver disease contributed a higher mortality than those without chronic liver disease.^10,11^ Cirrhosis has been proved to be an important prognostic factor in TB patients. However, it is still unknown if TB, like other bacterial infections has an important impact on the mortality of cirrhotic patients. In this study, we used nationwide population-based database to enroll a large population of cirrhotic patients with and without new diagnosis of TB, The aims of this study were (1) to investigate the effect of TB on mortality in cirrhotic patients; (2) to identify the persistence of this effect.

## METHODS

### Ethical Statement

For studies with human subjects, this study was initiated after approval by the Institutional Review Board of the Buddhist Dalin Tzu Chi Hospital in Taiwan (B1010410). Since all identifying personal information was stripped from the secondary files before analysis, the review board waived requirement for written informed consent from the patients involved.

### Database

In 1995, Taiwan started the National Health Insurance Program. Currently, the National Health Insurance Bureau (BNHI) covers more than 99% of the Taiwan population. All medical records from all contracted medical institutions must be provided to the BNHI for medical payment. In according to the regulations governing the review of the medical services, the BNHI reviews reimbursement claims filed by contracted medical institutions and screens the type, volume, quality and appropriateness of medical services provided under the National Health Insurance Program. These medical records are established as a database, the National Health Insurance Research Database (NHIRD), which is maintained by BNHI. The dataset in this study is from this database, which includes all International Classification of Diseases, 9th Revision, Clinical Modification (ICD-9-CM) codes of the hospitalized patients in Taiwan. The NHIRD research committee approved the use of this database to perform this study (agreement number 101516). The files from NHIRD did not include the patients’ and their health care providers’ private information.

### Study Sample

In this retrospective study, we searched the patients discharged between January 1, 2007 and December 31, 2007 with diagnostic codes for cirrhosis (ICD-9-CM code 571.5, or 571.2). Because the etiologies of cirrhosis were very different in young and adult cirrhotic patients, the patients < 30 years old were excluded. The patients with biliary cirrhosis (ICD-9-CM code 571.6) also were excluded. The patients with incomplete or missing basic data in the database were also excluded. In all cirrhotic patients, the patients with new diagnostic codes for TB (ICD-9-DM code 010.0–018.96) were enrolled.

In order to regress the effect of TB on the mortality of cirrhotic patients, we selected the factors related to the mortality of cirrhotic patients as comorbid medical factors, including alcoholism (ICD-9-CM codes 291, 303, 305.00–305.03, 571.0–571.3), hepatocellular carcinoma (HCC) (ICD-9-CM code 155.0), esophageal variceal bleeding (EVB) (ICD-9-CM code 456.0, 456.20), ascites (ICD-9-CM code 789.5, or procedure code 54.91), hepatic encephalopathy (HE) (ICD-9-CM code 572.2), renal function impairment (RFI), and bacterial infections. The patients with RFI were defined as those with diagnostic codes related to RFI (ICD-9-CM code 584, 585, 586, 572.4, or other procedure codes relate to renal failure).^12^ The bacterial infections included pneumonia (ICD-9-CM code 481–487, without 484),^13^ liver abscess (ICD-9-CM code 572.0), empyema (ICD-9-CM code 510), cellulitis (ICD-9-CM code 681 or 682), necrotizing fasciitis (ICD-9-CM code 728.86), central nerve system infection (ICD-9-CM code 324 or 320), sepsis (ICD-9-CM code 038, 020.0, 790.7, or 112.81),^14^ infective endocarditis (ICD-9-CM code 421), urinary tract infection (ICD-9-CM code 590.1, 595.0, 595.9 or 599.0),^15^ biliary tract infection (ICD-9-CM code 576.1, 575.0, 574.00, 574.01, 574.30, 574.31, 574.60, 574.61, 574.80, 574.81), septic arthritis, (ICD-9-CM code 711), perianal abscess (ICD-9-CM code 566), and spontaneous bacterial peritonitis. Spontaneous bacterial peritonitis was defined as a patient with the ICD-9-CM diagnosis codes with 567.2, 567.8, or 567.9, and without the procedure codes for the abdominal surgery.^16,17^ In addition to the diagnostic codes mentioned above, we checked all diagnostic codes of the enrolled patients to confirm if the patients have other bacterial infections.

The comparison cohort consisted of the cirrhotic patients without TB. To avoid the interference of measured confounding factors, we performed one-to-ten case-control match on propensity score matching (PSM) that was obtained from logistic regression of TB on age, alcohol-related, HCC, EVB, Ascites, HE, RFI, bacterial infection, and gender. Besides, the nearest-neighbor matching method was used in the propensity model.

### Statistical Analysis

The IBM SPSS Statistics package (IBM SPSS Statistics for Windows, Version 22.0. Armonk, NY: IBM Corp.) was used to perform the analyses in this study. PSM was performed using the PSM extension program developed by Felix Thoemmes for SPSS Statistics in 2011. The Chi-square test or Fisher's exact test was used to compare categorical variables. One-way ANOVA was used to compare continuous variables. In order to identify risk factors for the mortality, the proportional hazards Cox regression model was used to control for possible confounding factors. We presented hazard ratios (HR) with the 95% confidence intervals (CI) using a significance level of *P*-value < 0.05. The starting point to evaluate of the 30-day, 90-day, 1-year, and 3-year mortalities in the cirrhotic patients with and without TB was the date of admission in the enrolled hospitalizations. HR for the mortality was calculated for the comparison between TB and non-TB groups.

## RESULTS

A total of 434 cirrhotic patients with TB were enrolled in this study, including 347 (80.0%) with pulmonary TB, 4 (0.9%) with military TB, 72 (16.6%) with extrapulmonary TB, and 11 (2.5%) with both pulmonary and extrapulmonary TB. The comparison group consisted of 4340 selected cirrhotic patients without TB in the same period by PSM analysis. The mean age was 61.5 ± 14.2 years in non-TB group and 61.0 ± 14.8 years in TB group (*P* = 0.895). The demographic characteristics and comorbidities of the cirrhotic patients with and without TB are shown in Table 1. Through PSM, the baseline comorbid factors did not show statistically differences between TB and non-TB groups. The 30-day, 90-day, 1-year, and 3-year mortalities were 10.1%, 24.2%, 43.1%, and 63% in the TB group, and 7.9%, 15.5%, 31.2%, and 53.4% in the non-TB group.

**TABLE 1:**
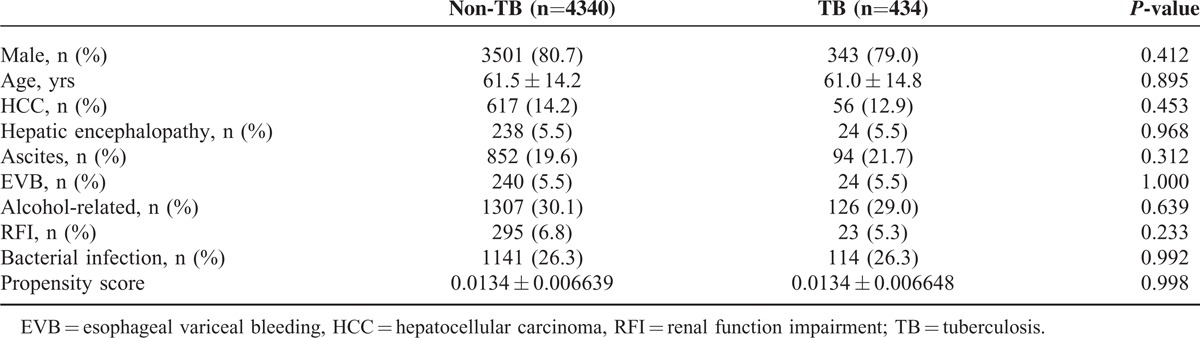
Demographic Characteristics for Cirrhotic Patients With or Without Tuberculosis (n=4774)

In a Cox proportional regression model, adjusted by age, gender and other comorbid disorders including HCC, EVB, ascites, alcoholism, RFI, bacterial infection, and HE, the HR of TB for the 90-day mortalities of cirrhotic patients was 1.62 (95% CI 1.32–1.99, *P* < 0.001). The HRs of other factors for the 90-day mortalities of cirrhotic patients were listed in Table 2. RFI, ascites and HE carried higher risk for mortalities in the cirrhotic patients.

**TABLE 2:**
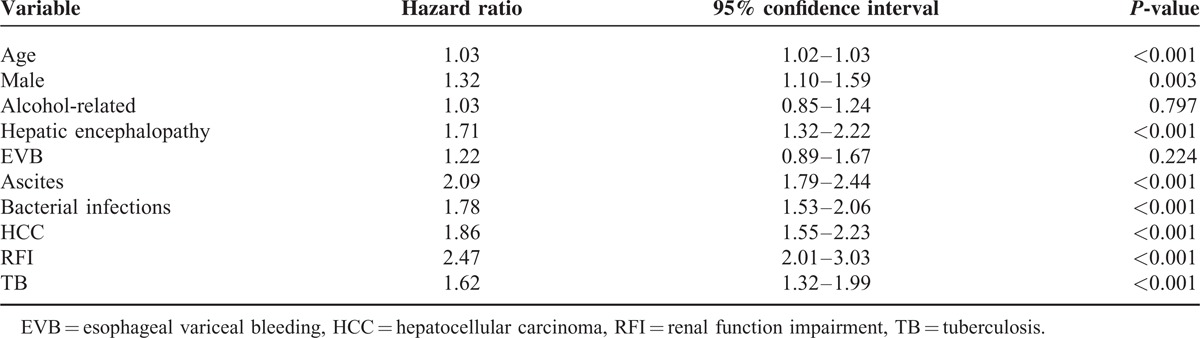
Adjusted Hazard Ratios of Risk Factors for the 90-Day Mortality in Cirrhotic Patients After Cox Regression Model

**FIGURE 1:**
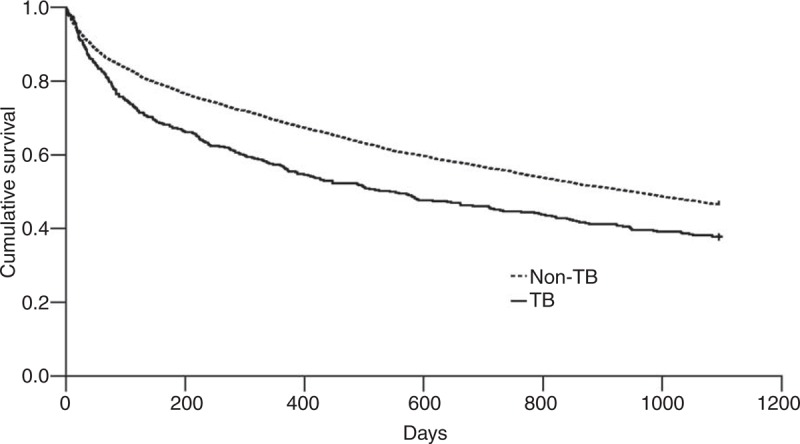
Cumulative survival plot for cirrhotic patients with and without tuberculosis After Cox regression model, the hazard ratios of tuberculosis (TB) for 30-day, 30 to 90-day, 90-day to 1-year, and 1 to 3-year mortalities were 1.33 (0.97–1.83), 1.91 (1.45–2.51), 1.46 (1.16–1.84), and 1.10 (0.88–1.37), compared to non-TB group. TB is a statistically significant risk factor for 30-day to 1-year mortality in cirrhotic patients.

In order to evaluate the early and late effects of TB on mortality, we calculated the 90-day mortality of the patients surviving more than 30 days, the 1-year mortality of the patients surviving more than 90 days, and 3-year mortality of the patients surviving more than 1 year. The 30 to 90-day, 90-day to 1-year, and 1 to 3-year mortalities were 15.6%, 24.9%, and 33.6% in TB group, and 8.3%, 18.5%, and 32.3% in non-TB group. After Cox proportional regression model adjusted by age, gender and other comorbid factors, the HRs of TB for 30-day, 30 to 90-day, 90-day to 1-year, and 1 to 3-year mortalities in cirrhotic patients were 1.33 (95% CI 0.97–1.83, P = 0.073), 1.91 (95% CI 1.45–2.51, *P* < 0.001), 1.46 (95% CI 1.16–1.84, *P* = 0.001), and 1.10 (95% CI 0.88–1.37, *P* = 0.408) (Table 3). We found that the effect of TB on the mortality of cirrhotic patients focused on the 30 day to 1 year after diagnosis of TB. Figure 1 showed the cumulative survival plot for cirrhotic patients with and without TB.

**TABLE 3:**
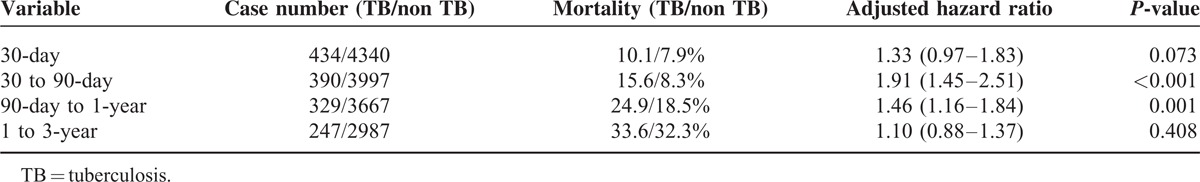
Adjusted Hazard Ratios of Tuberculosis for the 30-Day, 30 to 90-Day, 90-Day to 1-Year, 1 to 3-Year Mortalities of Cirrhotic Patients, Compared to Those Without Tuberculosis

## DISCUSSION

In 2007, the prevalence of TB was 111 per 100,000 population, and the incidence of TB was 32 per 100,000 population in Taiwan. Totally, 783 of 19,591 TB patients died in 2007 and the mortality was 4%. Our study showed the cirrhotic patients with TB had a 10-fold increase in 1-year mortality. This result was compatible with the data from India, where the mortality of TB patients with cirrhosis was 80/1000 population, much higher than those for the general population (0.5–2.3 per 1000 population).^18^ Our data also showed the cirrhotic patients without TB had 35.8% of 1-year mortality. This means that the mortalities of cirrhotic patients with TB were mostly attributed to cirrhosis itself.

Our study showed that the effect of TB on the mortality of cirrhotic patients focused on 30 days to 1 year after diagnosis of TB. The HR was especially higher in the interval of 30 to 90 days. We attributed this result no only to TB itself but also to the side effect of anti-TB chemotherapy. Previous study showed cirrhotic patients with TB still responded well to anti-TB chemotherapy.^19^ However, the incidence of hepatotoxicity induced by anti-TB chemotherapy in much higher in cirrhotic patients.^19,20^ This kind of hepatotoxicity usually occurs in the first 2 months of treatment, which can cause significant morbidity and mortality.^21,22^ This also can cause fewer cirrhotic patients with TB to complete the expected course of anti-TB chemotherapy that non-cirrhotic patients with TB.^19–22^ Accordingly, it is important to reduce treatment-induced hepatotoxicity for prolonging the survival of cirrhotic patients with TB. Presently, some ideas are developed for prevention of hepatotoxicity induced by anti-TB chemotherapy, including N-acetylcysteine,^23^ silymarin,^24^ and the herbal formulation of *Curcuma longa* and *Tinospora* cordifolia.^25^ However, their evidences have not been consensus. Early detection and prompt withdrawal of the offending drug is still the main way to correct anti-TB chemotherapy-induced hepatotoxicity. Monitoring liver function tests should be more frequently in cirrhotic patients receiving anti-TB chemotherapy, especially in 30 to 90 days after treatment.

It is very reasonable that TB has no effect on the mortality of cirrhotic patients 1 year after treatment. However, it is very interesting that TB itself has no effect on the first 30-day mortality of cirrhotic patients after diagnosis of TB. In clinical presentations, cirrhotic patients with TB have some characteristics, including low grade temperatures, marked weight loss, and more extrapulmonary involvement, but they still respond well to standard anti-TB chemotherapy.^19^ Previous study showed that a TB-related mortality rate was only 2.1%, and the median time from treatment initiation to death was about 23 days.^26^ Our study also showed about a 2.2% difference of 30-day mortality between TB and non-TB cirrhotic patients (7.9% vs 10.1%). However, this difference did not show statistically significant after regression model adjusted by other comorbid factors. This means that TB-related morality is very low under anti-TB chemotherapy within first 30 days.

Our study provides evidence that TB increases the mortality in cirrhotic patients; furthermore, the mortality risk focused on 30 day to 1-year after diagnosis of TB. Nonetheless, there were several limitations in our study. First, it was not possible to identify the Child-Pugh score according to the diagnostic ICD-9 codes in the database, although the Child-Pugh score in cirrhotic patients was not proved to be associated with the outcome in previous studies.^27,28^ Secondly, the etiology for cirrhosis in Taiwan is known mostly to be related to chronic vial hepatitis, like hepatitis B virus and hepatitis C virus.^29^ However, the exact etiology of liver cirrhosis could not be identified in this population-based study, even though there were 22.5% cirrhotic patients with diagnostic code of alcoholism. Finally, it was impossible to evaluate the severity and treatment course of TB in this database. Despite these limitations, this study is the first complete nationwide population-based study for identifying the effect of TB on the mortality of cirrhotic patient. In conclusion, the cirrhotic patients had increased 30 to 1-year mortality after diagnosis of TB.
